# Constructing personal networks in light of COVID-19 containment measures

**DOI:** 10.1186/s41118-021-00128-4

**Published:** 2021-08-26

**Authors:** Emanuela Furfaro, Giulia Rivellini, Elvira Pelle, Susanna Zaccarin

**Affiliations:** 1 Department of Statistical Sciences, Università Cattolica del Sacro Cuore, Milano, Italy; 2 Department of Statistics, University of California, Davis, USA; 3 Department of Communication and Economics, University of Modena and Reggio Emilia, Modena, Italy; 4 Department of Economics, Business, Mathematics and Statistics, University of Trieste, Trieste, Italy

**Keywords:** Personal networks, Lockdown, Relational vulnerability, Physical presence, Support

## Abstract

The policies for containing the spread of the SARS-CoV2 virus include a number of measures aimed at reducing physical contacts. In this paper, we explore the potential impact of such containment measures on social relations of both young adults and the elderly in Italy. We propose two ego-centered network definitions accounting for physical distance in light of the COVID-19 containment measures: the easy-to-reach network, that represents an accessible source of support that can be activate in case of new lockdown; the accustomed-to-reach network, which includes proximity and habit to meet in person. The approach used for constructing personal (ego-centered) networks on data from the most recent release of Families and Social Subject survey allows us to bring to the foreground people exposed to relational vulnerability. The analysis of the most vulnerable individuals by age, gender, and place of residence reveals that living alone is often associated with a condition of relational vulnerability for both the elderly and for young adults.

## Introduction

The outbreak of the SARS-CoV-2 virus caused, as of July 7, 2021, four million deaths worldwide (WHO, 2021). Within Europe, Italy was the first country to experience an outbreak, and it experienced one of the most severe ones : in early March 2020, Italy had already registered hundreds of deaths, being the European country with the largest death toll (WHO, 2020), and attracting worldwide attention (Boccia et al., 2020; Paterlini, 2020).

Starting at the end of February 2020, the Italian government announced some policies to contain the spread of the SARS-CoV-2 virus. These included social distancing, the prohibition of large gatherings and the closure of schools and universities, and they first targeted only those Italian territories heavily involved in the initial outbreak. A few days later, restrictions were further sharpened with the closure of all commercial and retail businesses except those providing essential services, and they were extended to the entire Italy, becoming, on March 9, 2020, the first country to implement a national quarantine amid COVID-19. Shortly after, other European and extra-European countries followed with very similar policies in an attempt to contain the spread of the SARS-CoV-2 virus.

Containment policies included a number of measures aimed at reducing physical contacts, considered an important factor in the SARS-CoV-2 transmission (CDC, 2021; Huang et al., 2020; Peeri et al., 2020). While the government’s efforts have been towards trying to lift restrictions on in-person school’s attendance, the reduction in physical contacts  and social gatherings have been prolonged, limiting personal networks of contacts. In fact, the available evidence demonstrates that the existing physical distancing recommendations, along with community use of well-fitting masks, adequate ventilation, and avoidance of crowded indoor spaces, prevent SARS-CoV-2 transmission (CDC, 2021).

The implications of the pandemic and the associated containment measures on demography, society, and economy are the object of study for many researchers who focus on direct (Dowd et al., 2020; Esteve et al., 2020) and indirect consequences (Arpino et al., 2020; Bonaccorsi et al., 2020; Luppi et al., 2020). Such implications also include changes in the network of social relations binding individuals to the people close to their everyday lives and in the availability of tangible and intangible resources they exchange.

The international research on the determinants of demographic micro-processes has convincingly shown the importance of including in the analysis not only the macro-, but also the meso-level, represented by the “social space” of individuals. The social space takes shape in the relations inside the (immediate or extended) family, and with friends, coworkers, or neighbors. It represents a resilience (anti-frailty) tool that can activate a protective network, stimulating the ability to adapt to and bear difficulties (Amati et al., 2017; Rosina and De Rose, 2017). The relevance of this level is also given by the influence that individuals have on others’ decision-making processes and behaviors through the transfer of their subjective perceptions of values and through their availability as a resource to pursue desired goals (Amati et al., 2015; Vikat et al., 2007). Moreover, the network of relationships, and especially its composition, is a reliable indicator of the sources, the quantity, the quality, and the types of support individuals have access to (Dykstra, 2007). In this context, the changes in social relations induced by the restrictions may compromise the possibility of collaborating and of feeling emotionally and socially supported, thus reducing the ability to cope with unfavorable events and with situations of stress.

COVID-19 is characterized by a strong age-dependence in mortality (Istat, 2020c; ODriscoll et al., 2021; Sasson, 2021). Focusing on the elderly population, recent studies on active aging have argued the importance of considering social cohesion and social support as a form of a wider social engagement and a stronger sign of an active lifestyle, underlying the importance of social relations in the aging process (Adams et al., 2011; Dykstra, 2007; Istat, 2020a; Pelle et al., 2021; WHO, 2002). In countries—such as Italy—characterised by strong solidarity between generations, the role of the elderly is also often associated with childcare activities and playing a central role in support (Mencarini and Solera, 2015). In Southern Europe and in Italy in particular, there is a large amount of interaction between older adults and younger individuals (Balbo et al., 2020). This is coupled with a high prevalence of inter-generational co-residence among older adults, suggesting on the one hand a potential higher vulnerability to age-sensitive epidemics and on the other hand potential larger social consequences (Balbo et al., 2020).

Furthermore, the literature highlights that in situations of stress, pressure, and uncertainty, social relations and social support structures may change in composition and size. The COVID-19 pandemic undoubtedly represents one of these shock events, given the transformations in everyday life, in the way of living social relations, in work habits, and in the behavior toward community life. During the pandemic, the network of relationships has hence been influenced by both the containment measures, and the general situation of emergency and uncertainty. This paper contributes to the recent discussion on the consequences of the containment measures by focusing on individual relationships.

Building upon the literature on personal (ego-centered) networks, on the basis of the most recent information on composition and characteristics of social networks in pre-pandemic time in Italy, we explore the potential impact of the containment measures on social networks. Since social networks are sources of support, often requiring geographical proximity to allow physical interactions and in-person contacts, we describe the changes in size and typology of social networks in light of the restrictions imposed during the pandemic. We will also identify different levels of vulnerability that individuals may suffer in their relational and support spheres. On the basis of the age-sensitive characteristics of COVID-19 as well as the strong inter-generational interactions still persisting in the Italian population, the analysis is carried out on two specific age groups: individuals aged 18–34 living alone or as a partner in a couple with or without children, with no other family (or non-family) members, and individuals aged 65 and over, living alone or as a partner in a couple without cohabiting children, and with no other family (or non-family) members. The social networks of individuals in these two specific living arrangements are composed by others from outside, allowing a clear picture of the sources of support they can mobilize if restrictions to social contacts and mobility are in force.

The remainder of the paper is organized as follows. "Social relations during the national lockdown of Spring 2020 in Italy" section discusses results from the most recent available surveys on relational aspects during the pandemic carried out in Italy and in other European countries. "Ego-centered network approach to studying social relations" section describes the Family and Social Subjects survey (FSS), the primary Italian data source for building the networks of contacts individuals (egos) entertain with others. Adopting an ego-centered network design with the most recent data from the pre-pandemic period, it is possible to evaluate the impact of the containment measures on individual contacts and sources of support. Results based on two different hypotheses of social interactions are presented and analyzed in "Ego-centered network construction in light of COVID-19" section, with a focus on individuals with a higher risk of relational vulnerability in case of a new emergency. "Concluding remarks" section presents concluding remarks.

## Social relations during the national lockdown of Spring 2020 in Italy

The final containment measures imposed on March 11, 2020, included a number of measures aimed at restricting physical contacts, which is a necessary condition for SARS-CoV-2 transmission (Huang et al., 2020; Peeri et al., 2020), while promoting social distancing and staying at home. In particular, the Italian government closed all schools, universities, non-essential workplaces, sports facilities, and commercial and retail businesses except those providing essential services, prohibited public events, non-essential travel, and any non-essential movement from home, including visits to family and friends, and strictly limited outdoor physical activity.^1^ These restrictions have pushed the population to change its routine as well as the way in which individuals live their relations with relatives, friends, acquaintances, and neighbors.

Several surveys have been carried out in Italy to study relational aspects during the first phases of the COVID-19 pandemic. In this section, we report the main results of those surveys that we believe are useful for motivating our research and interpreting our analyses.

Survey results presented in June 2020 in the Annual Report of the Italian National Institute of Statistics (Istat) highlight that during the March–April 2020 lockdown most people did not receive any visits or go out, respecting the restriction measures (Istat, 2020b). The report shows a cohesive and positive familiar environment that helped people cope with the uncertainty related to the health emergency. Relationships with cohabiting people are described as very good, as good as before the lockdown or even better, with most people being happy to entertain household members, depicting familiar relations and affections as shelters and sources of serenity during a time of uncertainty and fear. Perceiving the household environment as very good helped individuals live positively and constructively highlighting the importance of familiar ties. Relations with non-cohabiting people moved to a “virtual ground”, a change eased by the increasing use of technology observed in the last years.^2^ In particular, most Italians dedicated their spare time to cultivating their personal relationships. Most people spoke on the phone or through video calls with their relatives and friends, with more than one out of two dedicating more time than usual to this activity.^3^ Some people also reported playing games online with friends, suggesting again a translation of activities from physical to virtual. These results suggest that the way of living relationships changed, but frequency of contacts did not necessarily decrease. However, the loss of physical contact may have translated into a loss of support for those who were used to receiving instrumental support through a physical presence, such as families who counted on grandparents for the care of children or elders who required daily care.^4^

Other surveys have focused specifically on the changes in contacts that occurred during the COVID-19 pandemic (Arpino et al., 2020; Del Fava et al., 2020). From April 14 to April 24, 2020, Arpino et al., (2020) conducted an online survey of 9186 individuals aged 18 or older living in Italy, Spain, and France. Information spanned key domains of personal life, including type of relationships (with a focus on inter-generational relationships) and frequency of contacts. Preliminary findings showed that there was a large decrease in physical contacts, with people living in Italy and Spain experiencing higher reductions. Moreover, inter-generational physical contact was reduced particularly among younger adults, probably due to the wish to avoid contacts of grandchildren with older people. On the other hand, non-physical contacts have increased in all three countries, including contacts with non-relatives, other relatives, and all types of relationships. The survey also allows the study support received. The preliminary results show that a higher proportion of younger respondents reported having received more emotional and financial support compared to older groups.

Del Fava et al., (2020) also launched a cross-national online survey, the COVID-19 Health Behavior Survey (CHBS). Participants were recruited between March 12 and April 12 2020 via targeted Facebook advertisements for a total of 53,708 participants. Participants were asked to report the number of social contacts they had on the day before they completed the survey. The focus of the survey was epidemiological, aiming to examine the implications of reduced contacts on the spread of the coronavirus. Their findings hence provide evidence on the reduction in the number of contacts that occurred in the population, but they do not allow further study of the types of relationship or the people involved.

Given the relevance of the change in social contacts amid COVID-19, at the beginning of June 2020, the Survey of Health, Ageing and Retirement in Europe (SHARE), which collects longitudinal data on the elderly population in Europe, started fieldwork of the SHARE Wave 8 COVID-19 survey. The collected data—available since the end of December 2020—allow the study of different domains of the older adults’ lives during the pandemic, including social networks and changes in personal contacts with family and friends, help given and received, and personal care given and received. These ad hoc surveys designed during the pandemic are aimed at quickly providing information on number of contacts and how social relations have changed throughout the pandemic. However, they do not always allow for an extensive and detailed study on personal networks of relationships.

In this paper, we combine knowledge of the containment measures, the results provided by the above-mentioned studies, and the most recent data collected on personal networks through large-scale official surveys with the aim of elaborating new perspectives on the characteristics of personal support networks. These perspectives are mainly based on a novel use of relational information collected in the pre-pandemic period. The COVID-19 pandemic experience suggests the elaboration of new hypotheses for the construction of realistic personal support networks with the aim of identifying groups of individuals at risk in case of a new emergency.

## Ego-centered network approach to studying social relations

Data on contacts and social relations that individuals entertain with others are often collected through large-scale surveys.^5^ In these surveys, an ego-centered network design is adopted for data collection by asking the respondent (ego) to list the names/roles of people to which they are related (alters) in some ways (Crossley et al., 2015; Marsden, 2011; McCarty et al., 2019; Perry et al., 2018). Alters may include a variety of people, such as partner, parents, children, siblings, friends, neighbors, and colleagues. Further information on the alters’ characteristics (age, gender, place of residence, etc.) can also be collected to explore the characteristics of individual relations (ties) between ego and alter. Information on ties can also be added, such as the frequency, duration, and intensity of the relationship.

With a focus on Italy, the Family and Social Subjects (FSS) survey carried out by the Istat (https://www.istat.it/it/archivio/185678) represents the primary statistical source to reconstruct different types of ego-centered networks (Amati et al., 2015, 2017; Pelle et al., 2021). The FSS is based on a wide probability sample, allowing detailed network analyses in specific groups (by age, by living arrangements, etc.) of the population. In particular, the FSS survey asks for the presence of not-cohabiting siblings, children, and grandchildren (limited to a maximum of three, with grandchildren asked only of respondents who are at least 25), parents, and grandparents (asked only of respondents who are younger than 50), as well as the frequency of face-to-face contacts^6^ and residential proximity^7^ of siblings, children and grandchildren, and parents. An additional section collected information on the presence and, if any, type and number of other not-cohabiting relatives that respondents “are close to” or “to whom they can count on”, and on the presence and number of friends and neighbors respondents “can count on if necessary”. Besides the frequency of phone calls with not-cohabiting siblings, children and grandchildren, parents, and grandparents, in the last FSS edition, carried out in 2016, the frequency of video calls and messages (through sms, WhatsApp, email, social networks) was investigated as well the frequency of face-to-face contacts with friends, using the same answer categories proposed for siblings, children, etc.

By combining this information, networks of contacts, potential support networks and social support, networks are defined. With data from the FSS 2003, Amati et al. (2015) studied the “Potential Support ego-centered” (PSE) networks and the “Effective Support Ego-Centered” (ESE) networks of young adults aged 18-34 who are single or live with a partner. The PSE network was defined as the set of non-cohabiting people (along with their role relations) who can be a possible source of support to the respondent. Amati et al. (2015) assumed that alters with whom respondents entertained frequent contacts (“at least once in a week”) and were living nearby (even in a different municipality but not farther than 16 km) could be reliable potential supporters. The two conditions were thought to be a credible ground for the emergence of support ties. In particular, the assumption of geographical proximity was motivated by results on spatial and network analyses (Doreian and Conti, 2012) which showed that spatial proximity and location can influence the formation and nature of social ties. Moreover, certain forms of instrumental support (child and medical care, adult assistance, housekeeping, providing meals, etc.) can be better provided if proximity as well acquaintance hold. However, due to the in-place restrictions, geographical proximity and frequency of contacts assumed different values. In fact, the former was no more a condition to ease the provision of help and the latter became virtual. Istat (2020) confirms that, due to the lockdown, most Italians did not visit other people, and most people dedicated more time than usual to phone or video calls with relatives and friends.

The same PSE definition was adopted with the FSS 2009 to build the potential support network of individuals aged 18-44 living as couples to analyze the probability of receiving support from alters controlling for the social network characteristics (Amati et al., 2017).

Pelle et al. (2021) focused on individuals aged 65 and over and built the elderly’s ego-centered network of contacts to be considered as an explanatory variable in studying the support provided by older people, shedding new light on the topic of active aging in Italy.

The approach previously described shows another important advantage: it allows us to bring in the foreground people exposed to “relational” vulnerability. According to Ranci (2010), the “vulnerability identifies a situation that is characterized by a state of weakness which exposes a person (or a family) to suffering particularly negative or damaging consequences if a problematic situation arises.” If social vulnerability includes aspects connected to financial situation, housing conditions, employment, and management of care for children and dependent persons, the relational vulnerability focuses more on the social space of relationships, which is recognized as a resilience tool for most people. Relational vulnerability can be characterized by some basic elements: (a) the lack of available others to whom one can turn if needed; (b) a degree of dissatisfaction with available support; (c) the lack of strong or weak ties; (d) perception of loneliness. This vulnerability does not necessarily identify trajectories of grave loneliness or states of need but a high degree of exposure to serious damage. That is particularly true in cases of large-scale disasters—such as pandemics of infectious diseases, terrorist attacks or natural disasters—causing a significant loss of lives, property damage, and adverse social and economic impact (Chau et al., 2014). Usually in such situations, the main concern is the identification of frail individuals, who—regardless of the differing definitions of frailty—show physical, cognitive/psychological, nutritional, and social traits, as well as aging and disease, that are common contributing factors of frailty. Focusing on the COVID-19 pandemic, recent research has shown that frailty, more than age or co-morbidity, is associated with in-hospital mortality and a decreased probability of being discharged from the hospital (Hägg et al., 2020).

Building on the above literature and using relational data from the FSS 2016 edition—the most recent available data for the pre-emergency period—in the next section we propose different hypotheses to build relational contexts in which individuals could be embedded (ego-centered networks), taking into account the most recent months of COVID-19 restrictions with respect to physical distancing. From the resulting ego-network types, it is possible to derive the size and the characteristics, as well as level of relational vulnerability, of individuals in need of help in case of a new emergency. Since the analysis of social networks cannot disregard the ages of individuals and their life courses—even more in an age-sensitive pandemic situation—our analysis focuses on two different target populations: individuals aged 18-34 living alone or as a partner in a couple with or without children, and with no other family (or non-family) members, and individuals aged 65 and over, living alone or as a partner in a couple without cohabiting children, and with no other family (or non-family) members.

The ego-centered network characteristics of these target populations are of particular interest since the literature has often considered youth and the elderly as vulnerable categories (see, among others, Bost et al., 2002; Sherman et al., 2013), needing resilience tools to adapt and to bear difficulties or major, distressing events, such as childbirth, the managing of work and care, or the aging process. Elderly people are indeed generally more vulnerable of other population groups and need additional care and services in both pandemic and non-pandemic times. Elderly people are a heterogeneous population (the aging process itself is highly diverse and context-dependent) as well, with different levels of vulnerability regarding also types of personal networks. On the other hand, young individuals living alone or in a couple in the first stage of family formation are an interesting age group to analyze since their social behavior as well as their sources of support (e.g., from their parents or grandparents) can dramatically change due to emergency restrictions, revealing an unexpected frailty in everyday life if they cannot count on the presence of close alters.

## Ego-centered network construction in light of COVID-19

As discussed in the previous section, we use data from the latest edition of the FSS survey, carried out in 2016. Young adults aged 18-34 are the 20.5% (${n}=5084$) of the total Italian population in the survey (${n}=24{,}753$, unweighted data), while individuals aged 65 and over are the 27% (${n}=6771$). Our target groups represent the 22.6% (${n}=1148$), and the 75% (${n}=5085$), respectively for the young adults and the elderly in the FSS 2016. Main socio-demographic characteristics by living arrangement for the two age groups are reported in Table 1, where by “single” we mean people who live alone, and by “in couple”, we mean people who live with their partner, without children when considering the elderly and either with or without children when considering young adults.

**Table 1:** Socio-demographic characteristics, % (FSS 2016, unweighted data)

	Single	In couple		Single	In couple
	(${n}=400$)	(${n}=748$)		(${n}=1851$)	(${n}=3234$)
	34.8	65.2		36.4	63.6
(a) Young adults (${n}=1148$)			(b) Elderly (${n}=5085$)		
*Gender*			*Gender*		
Male	60.2	51.2	Male	28.6	52
Female	39.8	48.8	Female	71.4	48
*Age*			*Age*		
18-24	16.2	7.6	65-74	33.3	55.3
25-34	83.8	86.4	75+	66.7	44.7
*Health*			*Health*		
Good	92.9	94.5	Good	30.5	39.4
Fair	5.6	4.8	Fair	42.5	42.1
Bad	1.5	0.7	Bad	27	18.5
*Territorial area*			*Territorial area*		
North	45.5	50.3	North	46.1	45.3
Center	19.3	15.1	Center	15.7	18.3
South/Islands	35.2	34.7	South/Islands	38.2	36.4
*Place of residence*$^*$			*Place of residence*$^*$		
Metropolitan area	16.5	15.4	Metropolitan area	16.9	17.8
$\le 10{,}000$ inhab.	36	43.4	$\le$10,000 inhab.	38.4	37.4
$>10{,}000$ inhab.	47.4	41.2	>10,000 inhab.	44.7	44.8
*Education*			*Education*		
High	30	20.2	High	6	6.7
Medium	53	51.3	Medium	17.5	20.5
Low	17	28.5	Low	76.5	72.8
*Income*			*Income*		
Self-employed	16	12.7	Pension	88.5	80.6
Employed	62.5	63.9	Other	11.5	19.4
Other	21.5	23.4			
*Cohab. children*					
At least 1	–	64.3			

* Place of residence is recorded in three categories: metropolitan areas, municipalities up to 10,000 inhabitants, and municipalities beyond 10,000 inhabitants. The category “metropolitan area” includes the big city at the center of the metropolitan area (Turin, Milan, Venice, Genoa, Bologna, Florence, Rome, Naples, Bari, Palermo, Catania, Cagliari) and the municipalities within the borders of the area

Looking at young adults, about 60% of singles are men; both singles and couples are mostly aged 25-34 and have reached a good (medium or high) degree of education. As expected, almost all reported being in very good health. All told, 78.5% of singles and 76.6% of partners receive their main source of income from a job, while for 21.5% of young singles and 23.4% of young partners, the income is from a different source (maintenance/allowance).^8^ Conversely, looking at the elderly, more than 70% of singles are women. This is not surprising, considering the well-known gender differences in terms of health and life expectancy. Looking at health conditions, elderly adults in a couple appear to be healthier than singles. Among the elderly, 88.5% of singles and 80.6% of those in a couple receive a pension. Almost half of young adults and the elderly live in the North of Italy, while only around 16% of young adults and around 17% of the elderly live in a metropolitan area.

With the aim of exploring the potential impact of the containment measures on social relations of both young adults and the elderly, we propose two ego-centered network definitions accounting for physical distance in light of the recent—and potentially future—COVID-19 containment measures.

In particular, we define a social network characterized by the physical presence of alters^9^ that live in the same municipality as ego. This network is based on the idea of drawing a picture of the real availability of “easy-to-reach” alters in a personal network, which may represent a possible source of support in case of a new lockdown. In this perspective, an alter is included in the personal network of an ego if he/she lives in the same municipality, regardless of the frequency of face-to-face contacts.

Information contained in the FSS survey allows us to check for the residential proximity of different types of alters, according to age group. More specifically, we can check for the presence of parents and siblings in the personal networks of young adults and for the presence of siblings, children and grandchildren in the personal networks of the elderly. Regarding neighbors, we included them among the alters when the respondents confirmed that they could count on them. Unfortunately, the FSS questionnaire does not provide information on residential proximity of friends; however, it provides information on the frequency of face-to-face contacts. We use this frequency as a proxy of residential proximity with friends, in the sense that we assume that if ego has frequent (at least once in a week) face-to-face contacts with friends, this means that they live close by. We are aware that this is a strong assumption and it may not hold in certain contexts. However, the existing literature, which indicates that in-person contact occurring more than once a week is strongly associated with short distances (below 5 miles, Mok et al., 2010), along with the questionnaire wording (“Do you have one or more friends to count on in case of need?”), suggests that this assumption is reasonable for most of the cases in our data. Moreover, since in each of the considered age groups at least 70% of the sampled individuals declared to meet their friends regularly at least once in a week, disregarding the role of friends would be negligent.

**Fig. 1:**
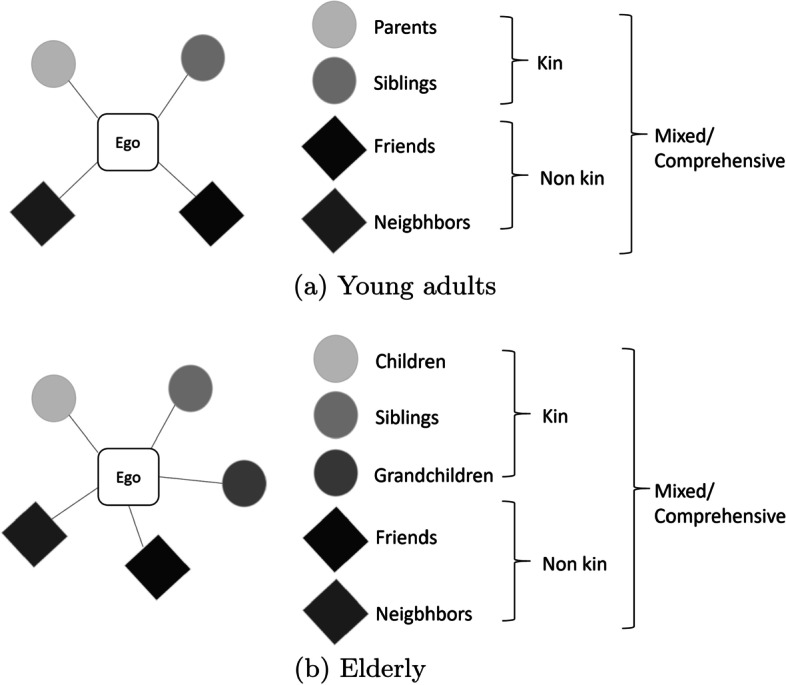
“Easy-to-reach” network by alter roles

We will refer to this personal network as an “easy-to-reach” network. The “easy-to-reach” network allows the presence of four different alter roles for young adults (parents, siblings, friends, and neighbors), and of five different alter roles for the elderly (siblings, children, grandchildren, friends, and neighbors).^10^ Combining the alter roles, we identify four network types (see Fig. 1): *Kin*, if alters are only related by kinship (parents/siblings or siblings/children/grandchildren);* Non-kin*, if a network is composed only by friends and/or neighbors; *Mixed*, if there is at least one alter belonging to the kin sphere and at least one friend or neighbor; and *Comprehensive*, if there is at least one alter from each alter role.

In order to deepen the study on the personal support network in which young adults and the elderly can be embedded in a possible new emergency situation, we decided to add information on the frequency of face-to-face contacts as an additional constraint for network construction. In particular, an alter is included in the personal network of an ego if he/she lives in the same municipality and has frequent face-to-face contacts with the ego (at least once in a week). As we added the habit to frequently meet and spend time together, we will refer to this personal support network as the “accustomed-to-reach” network. The rationale behind these network construction assumptions is that it is more likely that alters embedded in the “accustomed-to-reach” network can represent a possible source of support in situations of reduced possibility to travel (e.g. a new lockdown) with respect to the “easy-to-reach” network. Indeed, the habit to frequently meet may reduce the possible embarrassment the ego can feel in asking for help in case of need. It is worth noting that both networks may be helpful in the presence of extremely strict lockdown, such as the one in place in Italy in March–May 2020.

As mentioned in "Ego-centered network approach to studying social relations" section, the FSS questionnaire provides information on both the residential proximity and frequency of face-to-face contact only for the alters belonging to the kin sphere. Thus, we check for the residential proximity and frequent face-to-face contacts for parents and siblings to build the “accustomed-to-reach” networks of young adults, and for the residential proximity and frequent face-to-face contacts of siblings, children, and grandchildren for the elderly.

Conversely, assumptions about friends and neighbors are the same as in the “easy-to-reach” network.

Analogously to the “easy-to-reach” network, we combine the alter roles in order to identify the network types: *Kin, Non-kin, Mixed*, and *Comprehensive*.

In this framework, for both network definitions—“easy-to-reach” and “accustomed-to-reach”—we can identify the individuals declaring the absence of not-cohabiting people to share activities and resources (*No Alters* individuals) in order to recognize a condition of relational vulnerability that put them at risk of frailty, especially if they live alone.^11^

In the next section, we provide some descriptive results on the personal networks built following the above-described steps. Results in Tables 2, 3 and 4 (“easy-to-reach” network) and Tables 5, 6 and 7 (“accustomed-to-reach” network) are presented considering the two age groups (young adults or elderly) by living arrangements (single or in couple) and gender.

### The “easy-to-reach” network

The “easy-to-reach” network highlights the physical presence of alters. These may represent a source of support in situations of reduced possibility to travel. Table 2 shows the distribution of the number of different roles reported in Fig. 1 (i.e., three friends and three siblings count as two roles) in the young adults’ and elders’ social networks. These tables allow us to understand the heterogeneity of the alters roles, which is an important characteristics to take into account when studying social networks. In fact, different roles may be a source of different types of support. Regarding gender, in the observed sample, women seem to have a larger availability of alters with different roles but, when in couple, the gender differences are smaller. Focusing on age, the availability of alters in different role relations is similar between the two considered age groups. The group of physically isolated people is represented by those having no different role relations in their networks (row 1 of Table 2, Panel (a) e (b)). Generally, men seem more frequently physically isolated.

**Table 2:** Distribution of number of different roles—“easy-to-reach” network

	Single		In couple			Single		In couple	
	Males	Females	Males	Females		Males	Females	Males	Females
(a) Young adults					(b) Elderly				
0	12.9	7.5	13.7	11.7	0	14.6	9.2	9.8	10.8
1	22.8	24.5	19.5	24.5	1	25.0	19.8	17.1	18.3
2	28.2	22.6	34.8	31.6	2	27.8	29.3	25.8	27.0
3	22.4	27.0	18.6	19.6	3	19.7	24.8	26.7	25.2
4	13.7	18.2	13.4	12.5	4	10.0	11.6	14.6	13.6
					5	3.0	5.3	6.1	5.1

Table 3 reports network size by alter roles taking into account the number of alters in each role (i.e., three friends and three siblings count like six). Table 3 thus shows the mean and median number of parents, siblings, and friends,^12^, when focusing on young adults (Panel (a)), and the median and mean number of children, siblings, grandchildren, friends, when referring to the elderly population (Panel (b)). Focusing on young adults, the main observed difference is in the availability of friends. In fact, singles often have more friends to count on in case of need than couples have. The main gender differences are observed with regard to neighbors, with women having neighbors to count on more often. Looking at the elderly population, the average number of friends living nearby drops consistently both for women and men, in couple and single. Conversely, the number and frequency of first-degree relatives is similar between young adults and the elderly.

**Table 3:** Network size by alter roles—“easy-to-reach” network

	Single (males)			Single (females)			In couple (males)			In couple (females)		
	%	Mean (sd)	Median	%	Mean (sd)	Median	%	Mean (sd)	Median	%	Mean (sd)	Median
(a) Young adults												
Parents	49.4	0.9	0	56	1	1	49.3	0.9	0	47.3	0.8	0
		(0.93)			(0.96)			(0.95)			(0.93)	
Siblings	34.9	0.4	0	42.8	0.6	0	44.7	0.6	0	37.6	0.5	0
		(0.67)			(0.77)			(0.81)			(0.80)	
Friends	68.9	2.9	2	68.6	2.7	2	56.7	2.2	1	55.6	2.1	1
		(3.05)			(2.81)			(2.80)			(2.62)	
Neighbors	48.1		0	56.6		1	47.9		0	56.1		1
(b) Elderly												
Children	43.9	0.7	0	58.9	1	1	61.8	1	1	60.8	1	1
		(0.92)			(0.97)			(0.92)			(0.93)	
Siblings	38.9	0.6	0	37.4	0.6	0	44.7	0.8	0	40.7	0.7	0
		(0.92)			(0.92)			(0.98)			(0.97)	
Grandchildren	34.8	0.7	0	48	1	0	48.8	1	0	48.7	1	0
		(1.1)			(1.19)			(1.17)			(1.19)	
Friends	31.8	1.1	0	30.7	0.9	0	31.6	1.1	0	28.7	1	0
		(2.21)			(1.84)			(2.17)			(2.12)	
Neighbors	45.4		0	50.7		1	50.7		1	48.7		0

Table 4 illustrates the observed distribution of the “easy-to-reach” network types, along with the network mean size and standard deviation in parenthesis. Overall, the most common types among young adults are *Non-kin* and *Mixed*, whereas the elderly are more often embedded in *Kin* and *Mixed* networks. In particular, single young men are mainly embedded in *Non-kin* networks, with 4.3 alters on average. Single young women, who have a slightly larger presence of family members, are slightly more often embedded in *Mixed* networks, with 6 alters on average. In general, regardless of gender and living arrangements, the two most common types are either *Non-kin*, driven by the presence of friends, or *Mixed*, when the presence of friends is also coupled by the presence of kin. Focusing on the elderly population, regardless of gender and living arrangements, most people are embedded in either a *Kin* network or a *Mixed* network, denoting the constant presence of kin living in the same municipality. The mean size of the former is always higher than that of young adults since for the elderly, kinship includes grandchildren as well (see Fig. 1), but even possibly denoting that the whole family of their kids lives in the same municipality. Single elder males are found to be physically alone more often than any other group.

**Table 4:** Distribution of network types (mean network size and standard deviation)—“easy-to-reach” network

	Single			In couple		
	Males	Females	All	Males	Females	All
(a) Young adults						
No Alters	12.9% (0)	7.5% (0)	10.7% (0)	13.7% (0)	11.8% (0)	12.7% (0)
Kin	9.1% (2.3; SD 0.99)	10.1% (2.4; SD 1.15)	9.5% (2.4; SD 1.05)	14.8% (2.6; SD 1.24)	14.4% (2.7; SD 1.27)	14.6% (2.7; SD 1.3)
Non-kin	34.0% (4.3; SD 3.13)	30.2% (3.3; SD 2.67)	32.5% (3.9; SD 2.99)	26.9% (4.2; SD 3.29)	35.5% (3.7; SD 2.81)	31.3% (3.9; SD 2.03)
Mixed	30.3% (5.8; SD 2.62)	34.0% (6.0; SD 2.75)	31.8% (5.9; SD 2.67)	31.2% (5.1; SD 2.45)	25.8% (5.3; SD 2.36)	28.4% (5.2; SD 2.4)
Comprehensive	13.7% (9.2; SD 2.98)	18.2% (8.8; SD 2.62)	15.5% (9; SD 2.8)	13.4% (8.1; SD 2.39)	12.5% (7.8; SD 2.49)	12.5% (8; SD 2.4)
(b) Elderly						
No Alters	14.6% (0)	9.2% (0)	10.7% (0)	9.8% (0)	10.8% (0)	10.3% (0)
Kin	30.6% (3.1; SD 1.98)	34% (3.5; SD 1.98)	33.1% (3.4; SD 1.98)	32.1% (3.8; SD 2.13)	34.8% (3.7; SD 2.12)	34.4% (3.8; SD 2.13)
Non-kin	19.1% (2.6; SD 2.37)	15.7% (2.7; SD 2.47)	16.6% (2.6; SD 2.44)	13% (2.5; SD 2.38)	14.7% (2.7; SD 2.51)	13.8% (2.6; SD 2.45)
Mixed	32.7% (5.5; SD 3.0)	35.9% (5.2; SD 2.52)	35% (5.3; SD 2.66)	39% (5.6; SD 2.74)	34.6% (5.6; SD 2.79)	36.9% (5.6; SD 2.76)
Comprehensive	3% (10.1; SD 3.95)	5.3% (9.7; SD 2.57)	4.6% (9.8; SD 2.85)	6.1% (9.9; SD 2.91)	5.1% (10.2; SD 3.47)	5.6% (10; SD 3.16)

**Fig. 2:**
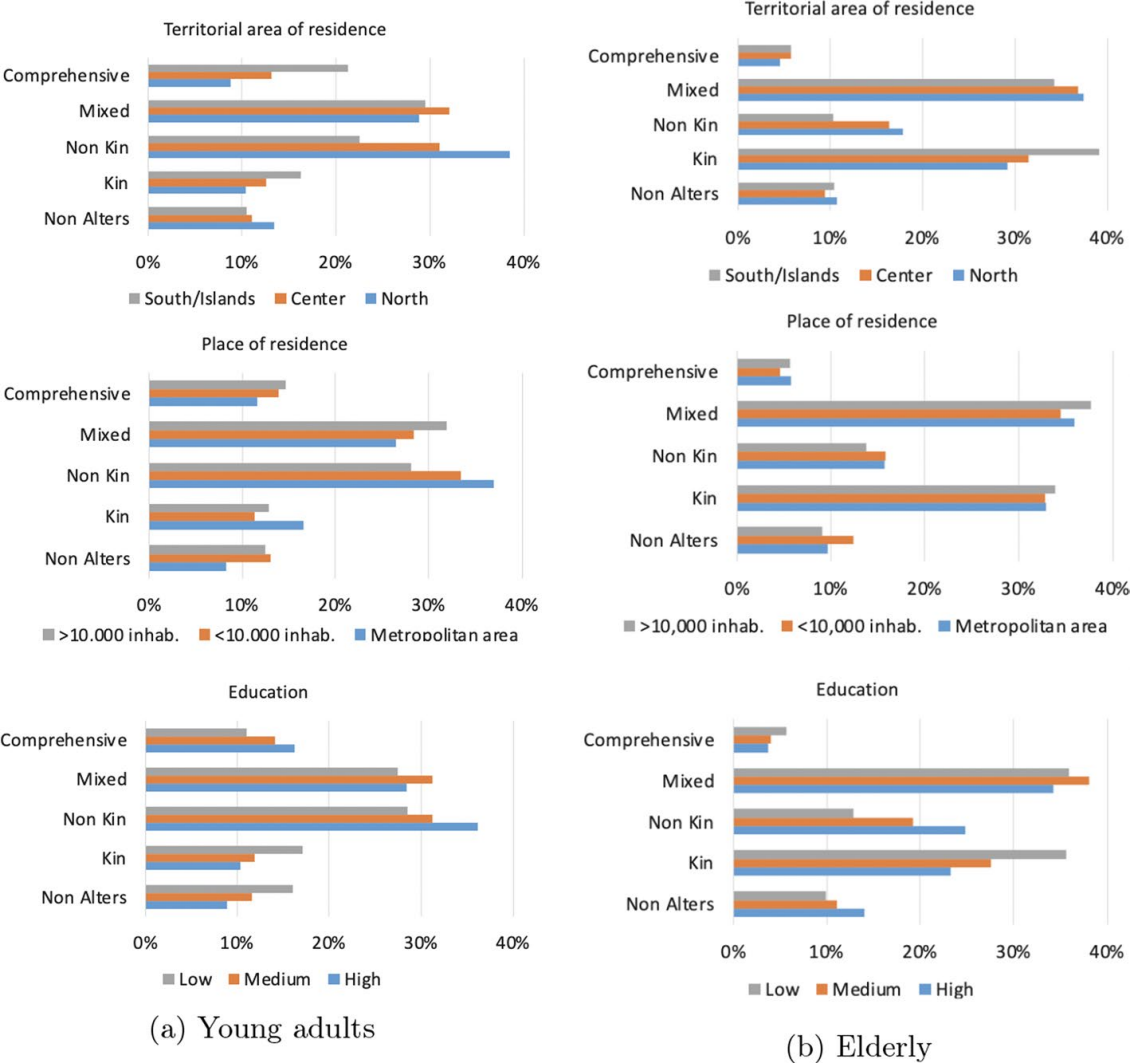
Distribution of “easy-to-reach” network types by territorial area, place of residence, and education

Last, Fig. 2 shows the distribution of network types by territorial area, place of residence, and education, among the two age groups considered. We did not divide by living arrangements since preliminary analysis did not highlight major differences. In particular, by looking at territorial area (top panel), it is easy to see the striking geographical differences among young adults, with the *Comprehensive* “easy-to-reach” type being mainly concentrated in the Southern regions and the *Non-kin* network type being more heavily present in the North. The opposite appears among the elderly, with the *Kin* type more present in the South. Both among young and the elderly the *Non-kin* type is more diffused among highly educated people, while it is reversed for the *Kin* type. *No Alters* networks and their relation with other individuals’ characteristics will be further discussed in "A focus on relational vulnerability: the case of *No Alters*" section.

### The “accustomed-to-reach” network

The “accustomed-to-reach” network highlights not only the physical presence of alters, but also the habit of meeting and spending time together. These most likely already are a source of support for the respondents, and, due to their physical proximity, they can keep representing an effective source of support in situations of reduced possibility to travel. Results for this network are related to the “easy-to-reach” network in the sense that we expect these networks to be “smaller” than the “easy-to-reach”. For instance, if a respondent had an empty “easy-to-reach” network, the “accustomed-to-reach” network will also be empty, while if the respondent had a non-empty “easy-to-reach” network because he/she did have some relatives living in the neighborhood, the “accustomed-to-reach” network may be empty if these relatives are hardly ever met.^13^

**Table 5:** Distribution of number of different roles—“accustomed-to-reach” network

	Single		In couple			Single		In couple	
	Males	Females	Males	Females		Males	Females	Males	Females
(a) Young adults					(b) Elderly				
0	14.5	9.4	15.6	12.5	0	17.6	11.1	12.4	13.2
1	24.1	27.0	20.0	24.5	1	27.6	23.1	19.6	20.9
2	29.5	25.8	35.1	31.6	2	30.2	31.8	28.7	29.2
3	20.3	21.4	17.3	19.8	3	15.9	20.3	22.5	20.8
4	11.6	16.4	12.1	11.5	4	6.8	9.9	12.4	12.4
					5	1.9	3.6	4.3	3.5

Table 5 shows the distribution of the number of different roles according the new network definition. In particular, all percentages of those who have an empty network (i.e., number of different alters $= 0$) increase, indicating that there is a subgroup of people—especially among males—that are not only physically, but also socially isolated. Similarly, the percentages of those whose network is composed by alters that have different roles decrease.

**Table 6:** Network size by alter roles—“accustomed-to-reach” network

	Single (males)			Single (females)			In couple (males)			In couple (females)		
	%	Mean (sd)	Median	%	Mean (sd)	Median	%	Mean (sd)	Median	%	Mean (sd)	Median
(a) Young adults												
Parents	44.4	0.8	0	47.2	0.9	0	47.1	0.9	0	46	0.8	0
		(0.92)			(0.95)			(0.94)			(0.92)	
Siblings	29	0.4	0	35.8	0.5	0	38.4	0.5	0	35.5	0.5	0
		(0.62)			(0.73)			(0.79)			(0.78)	
(b) Elderly												
Children	40.3	0.6	0	57.3	0.9	1	59.2	0.9	1	59.1	0.9	1
		(0.87)			(0.94)			(0.91)			(0.92)	
Siblings	27.6	0.4	0	24.7	0.4	0	28.7	0.5	0	26.9	0.4	0
		(0.80)			(0.78)			(0.83)			(0.83)	
Grandchildren	27.4	0.6	0	42.3	0.9	0	45.5	0.9	0	45.4	0.9	0
		(1.01)			(1.14)			(1.15)			(1.16)	

Table 6 is the counterpart of Table 3 (friends and neighbors are not reported here since the same assumptions of the “easy-to-reach” network hold for these alters). By comparing the two tables, we can highlight the subgroups that are not accustomed to leveraging their kin in the “easy-to-reach” network. Regardless of age, gender, and living arrangement, the major differences regard the frequency of physical contacts with siblings. The percentage of those who are accustomed to seeing their siblings drops in all groups. This is particularly evident if compared to the habit of seeing parents or children, in the young adults and elderly groups, respectively; instead, figures remain fairly consistent from the “easy-to-reach” network to the “accustomed-to-reach” network. This may indicate that people choose to live close to their parents or children with the goal of seeing them (possibly for providing or receiving support), while the proximity of siblings may be incidental. Grandchildren, if present, seem to entertain physical contacts with their grandparent, except for single men, who seem less accustomed to meeting with their grandchildren.

Table 7 outlines the distribution of network types. The major difference, with respect to the “easy-to-reach” network is the increase in the percentage of those who have *No Alters* in their network. The most common types are again *Non-Kin* and *Mixed* for young adults and *Kin* and *Mixed* for the elderly. The sizes are very similar compared to the “easy-to-reach” network, suggesting that the structure of the networks in the four non-empty categories remains stable, although there are a few less people who have heterogeneous networks composed both by kin and non-kin alters. The *No Alters* networks are, as already observed, more frequent, highlighting again a subgroup of people that, although it could potentially benefit from social interaction, does not. Trivially, the frequency of *Comprehensive* networks also decreases, highlighting a group of people who could potentially count on a very diverse network but in practice does not. This can be read along with the results on siblings derived from Table 6.

**Fig. 3:**
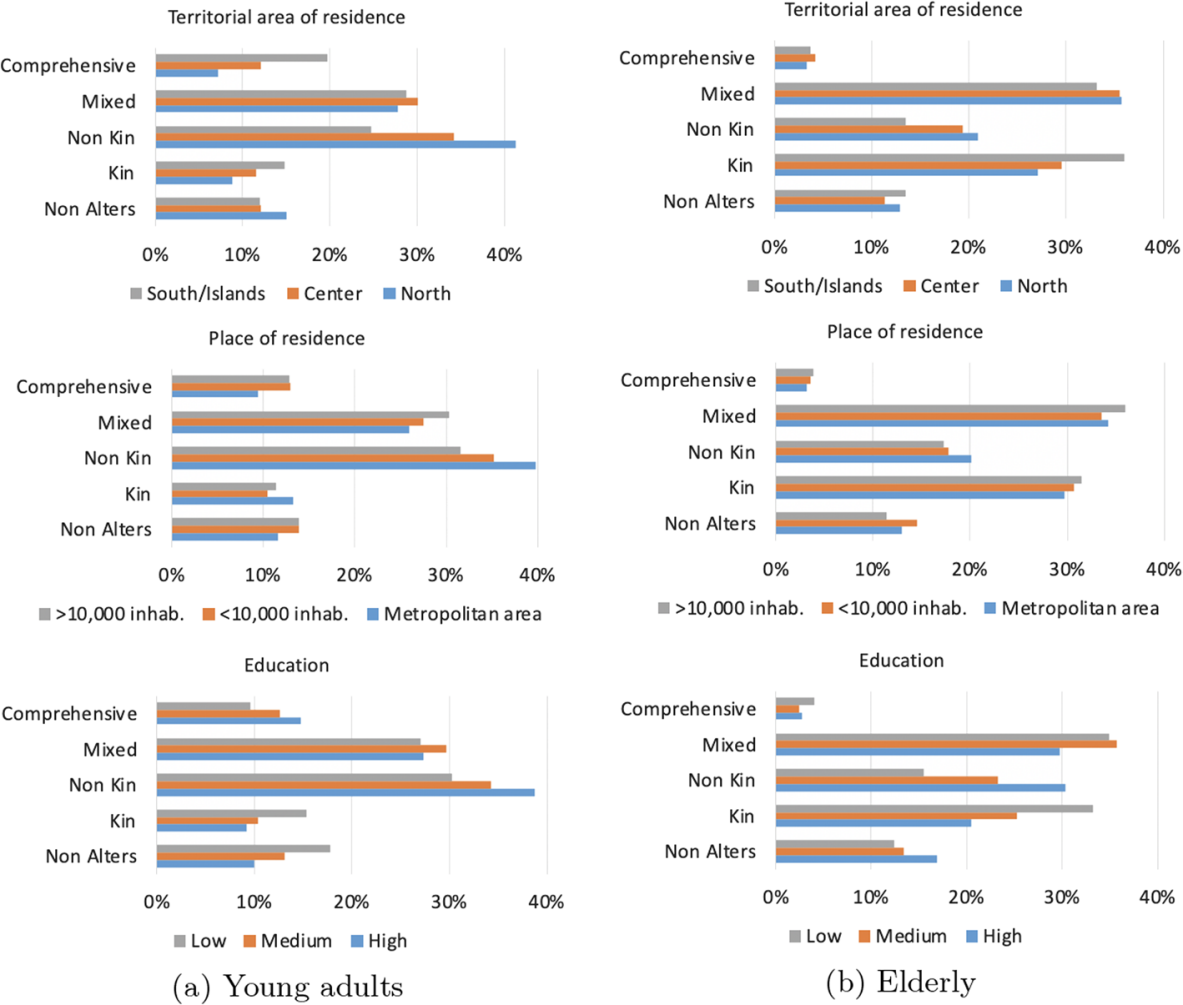
Distribution of “accustomed-to-reach” network types by territorial area, place of residence, and education

Last, similarly to "The “easy-to-reach” network" section, Fig. 3 shows the distribution of network types by territorial area, place of residence, and education. Similarly to the previous results, while in the North young adults are accustomed to meet with non-kin, Southern regions are characterised by the presence of a *Comprehensive* network type. Differences across education levels show that the *Non-kin* type is more present among highly educated individuals, both for young adults and the elderly.

**Table 7:** Distribution of network types (mean and standard deviation)—“accustomed-to-reach” network

	Single			In couple		
	Males	Females	All	Males	Females	All
(a) Young adults						
No Alters	14.5% (0)	9.4% (0)	12.5% (0)	15.6% (0)	12.5% (0)	14% (0)
Kin	7.5% (2.3; SD 0.96)	8.2% (2.1; SD 0.95)	7.8% (2.2; SD 0.95)	12.9% (2.6; SD 1.19)	13.6% (2.7; SD 1.25)	13.2% (2.6; SD 1.22)
Non-kin	37.8% (4.1; SD 3.04)	38.4% (3.5; SD 2.67)	38% (3.8; SD 2.91)	28.8% (4.1; SD 3.24)	35.8% (3.7; SD 2.81)	32.5% (3.9; SD 3)
Mixed	28.6% (6; SD 2.65)	27.7% (6; SD 2.71)	28.2% (6; SD 2.66)	30.7% (5.1; SD 2.41)	26.6% (5.3; SD 2.29)	28.6% (5.2; SD 2.53)
Comprehensive	11.6% (9.4; SD 3.06)	16.3% (9; SD 2.62)	13.5% (9.2; SD 2.83)	12% (8.4; SD 2.29)	11.5% (7.8; SD 2.56)	11.7% (8.1; SD 2.43)
(b) Elderly						
No Alters	17.6% (0)	11.1% (0)	13% (0)	12.4% (0)	13.2% (0)	12.8% (0)
Kin	27.6% (2.7; SD 1.78)	32.1% (3; SD 1.8)	30.8% (2.9; SD 1.8)	29.4% (3.4; SD 1.93)	32.4% (3.4; SD 1.94)	30.9% (3.4; SD 1.9)
Non-kin	22.5% (2.6; SD 2.37)	18.5% (2.6; SD 2.42)	19.7% (2.6; SD 2.4)	16.7% (2.7; SD 2.6)	17.3% (2.7; SD 2.56)	16.9% (2.7; SD 2.58)
Mixed	30.4% (5.3; SD 3.05)	34.7% (5.1; SD 2.5)	33.4% (5.2; SD 2.65)	37.2% (5.5; SD 2.81)	33.6% (5.5; SD 2.76)	35.5% (5.5; SD 2.79)
Comprehensive	1.9% (9.5; SD 3.89)	3.6% (9.4; SD 2.97)	3.1% (9.4; SD 3.1)	4.3% (9.3; SD 2.78)	3.5% (10.7; SD 3.5)	3.9% (9.9; SD 3.16)

### A focus on relational vulnerability: the case of *No Alters*

With FSS data, we can investigate relational vulnerability provided by at least two of the four basic elements mentioned in "Ego-centered network approach to studying social relations" section: the lack of available others to whom one can turn if needed (element a), which may reveal the absence of strong or weak ties (element c). Taking into account the different hypotheses on proximity and frequency vis-á-vis contacts with alters used to build the “easy-to-reach” and “accustomed-to-reach” networks, we focus on *No Alters* individuals among young adults (n=138 in the “easy-to-reach” network and ${n}=155$ in the “accustomed-to-reach” network, representing 12% and 13.5% of young adults, respectively) and the elderly (${n}=530$ in the “easy-to-reach” network, and ${n}=654$ in the “accustomed-to-reach” network, representing 10.4% and 12.9% of the elderly, respectively) to propose a criterion to identify groups of subjects at diverse levels of relational vulnerability, although sharing the same network type.

We first distinguish between the type of network identified, with the idea that those who show a *No Alters* type in an “easy-to-reach” network are at a critical lack of available nearby others to whom one can turn in times of need. The *No Alters* in the “accustomed-to-reach” network still exhibit a lack of available nearby alters, but they are considered less critical in terms of relational vulnerability. In fact, the conditions for alters inclusion are more strict since the frequency of contacts is also taken into account. But meeting each others is a clear sign of the real presence of relational ties—strong or weak—and in case of emergency an individual can activate these ties. Furthermore, the analysis of the relational vulnerability should not disregard the living arrangement and the stage of life course, strictly related to the groups of analysis.

Additionally, in this analysis, we consider the elderly as more vulnerable than young adults, particularly in case of external support for health-related reasons in non-pandemic time and—as cited above—the strong age-dependence in mortality of COVID-19. Moreover, for individuals in couple, the presence of a partner is recognized as protective, especially for the elderly. Conversely, among couples, their partners can be considered an adequate relational resource and bring the respondents sharing this living arrangement to declare not having any non-cohabitant people who can be a source of possible support. Therefore, individuals living in a couple benefit from relational ties with their partners. As a result of this, they are less vulnerable than the individuals living alone, regardless of the type of ego-centered network.

From the above reasoning, we then suggest a distinction of the relational vulnerability into three levels, as shown in Table 8. To be a *No Alters* single individual, regardless of stage of life course and type of network, is associated to the highest relational vulnerability. The presence of an “accustomed-to-reach” network is more protective, regardless of being single, in a couple, young, or elderly.

**Table 8:** Levels of relational vulnerability of *No Alters* in “easy-to-reach” and “accustomed-to-reach” networks

Level	Age groups	Network
Very critical	Elderly and young adults—single	“easy-to-reach”
	Elderly—single	“accustomed-to-reach”
More critical	Elderly and young adults—in couple	“easy-to-reach”
Critical	Young adults—single	“accustomed-to-reach”
	Elderly—in couple	
	Young adults—in couple	

There are other relevant demographic characteristics to check in order to better identify groups of individuals at risk in case of a new emergency: gender, age, context of residence, and education.

**Fig. 4:**
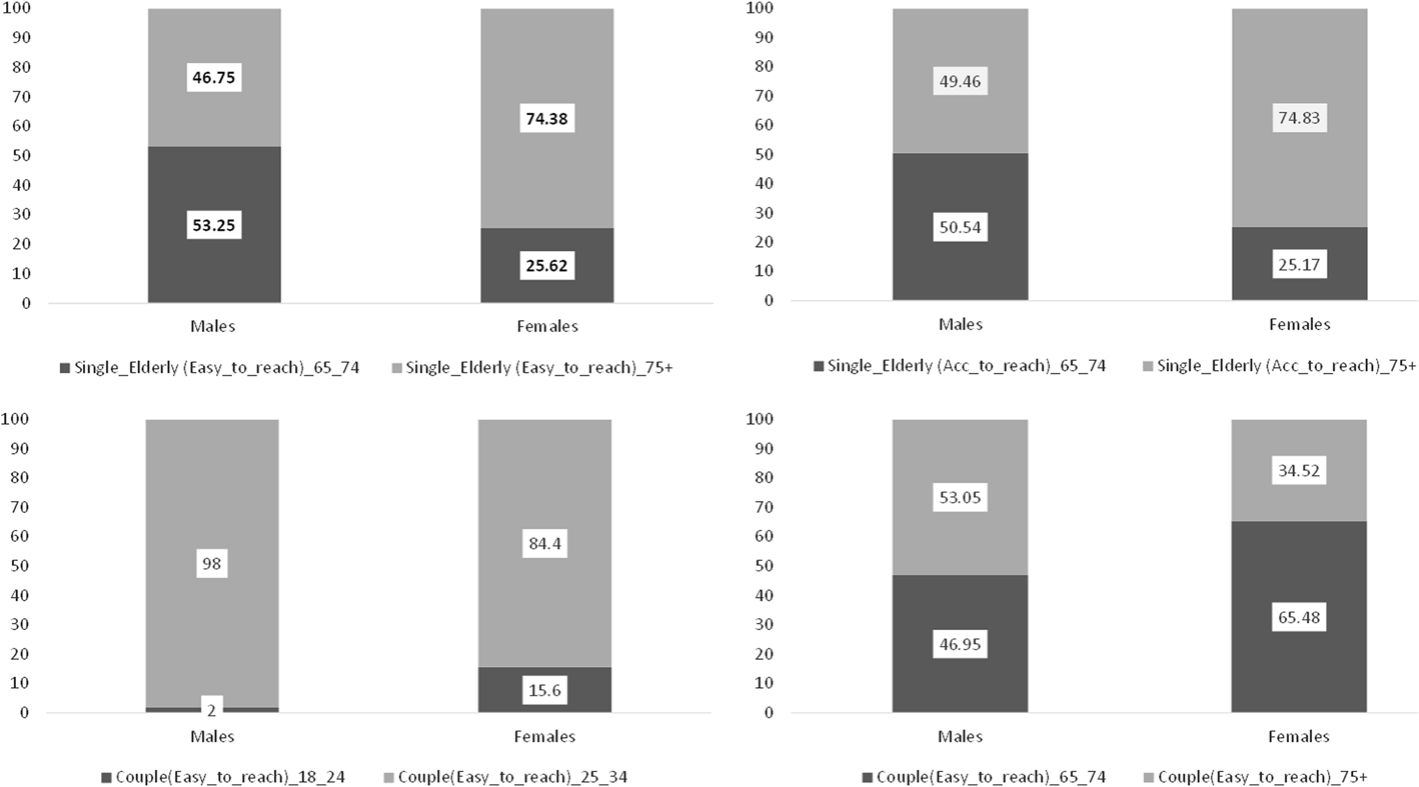
Individuals with a very critical and more critical relational vulnerability by gender and age class (%)

The following set of figures (see Fig. 4) shows a mixed gender-age effect among *No Alters* individuals^14^ with a “very critical” and “more critical” level of vulnerability. Among the single elderly, entering in an older age class (75+) coincides with an overturning in the incidence of those who are in a very critical situation by gender: 47% (male) versus 74% (female) and again—with “accustomed-to-reach”—49% (male) versus 75% (female), while in the younger age class we observe a reverse relationship (53% for male versus 26% for female and again 51% versus 25%). Among the single young adults, the highest relational vulnerability is observed only among the older age class (25-34), with no gender differences, different from the youngest couples (18-24), where there is a not negligible female incidence (16%). Among the elderly living in a couple, the situation appears up side down with respect to the elderly living alone: males (53%) in the oldest age class, more than females (35%) are classified in a critical relational situation, while the opposite is observed among the younger couples. Among elderly couples, males are on average older than females and the probability of observing females taking care of partners is higher than in the opposite case (males caregivers of females).

The recent pandemic experience has also revealed a high value of territorial data on different scales up to the municipality level. This is useful for monitoring the evolution of cases and deaths, but also for defining, managing, and assessing the policies introduced. During the pandemic the increase in mortality in Italy was characterized by a very high degree of heterogeneity at the territorial level (Blangiardo et al., 2020). We first checked for any significant difference in the distribution of *No Alters* individuals by territorial area, considering the level of relational vulnerability. However, the analyses did not show any significant pattern.

Nevertheless, focusing on the metropolitan area—seen as a relevant place of residence for monitoring and facing a risk situation—some interesting patterns emerged. Table 9 shows the percentage of *No Alters* individuals living in a metropolitan areas by gender and level of relational vulnerability.^15^ Among the elderly, the percentage of females relationally vulnerable is constantly lower than that of males; the opposite is observed among the young adults; moreover, as expected, young adults are less often in critical situations. In general the metropolitan area can be interpreted as quite protective with respect to isolation. In Table 9 we do not observe significantly high values; however, focusing on the highest percentages, we can recognize a worse condition associated with being single, elderly and male.

**Table 9:** Age groups of *No Alters* individuals living in metropolitan area by level of relational vulnerability, network and gender (%)

	Males	Females
Very critical		
Single elderly “easy-to-reach”	20.8	11.6
Single elderly “accustomed-to-reach”	20.4	14.3
Single YA “easy-to-reach”	3.2	8.3
More critical		
In couple elderly “easy-to-reach”	18.3	15.5
In couple YA “easy-to-reach”	14.0	13.3
Critical		
Single YA “accustomed-to-reach”	2.9	13.3
In couple elderly “accustomed-to-reach”	19.6	16.6
In couple YA “accustomed-to-reach”	19.3	14.6

With respect to economic vulnerability we do not dispose of enough informationl to capture that condition^16^, but the joint analysis between education level and relational vulnerability by type of network showed that the highest level of relational vulnerability (very critical) is associated with a low education level, especially for the elderly.

## Concluding remarks

The SARS-CoV-2 pandemic has put a strain on social and economic organization worldwide. The implications of the interventions adopted to contain the spread of the disease and the uncertainty of infection exposure have profoundly changed individuals’ lifestyles and behaviors, especially in the initial phases in 2020, worsening previous inequality gradients. In particular, the adopted large-scale lockdown as well as the further, although lighter, local-scale social distancing measures have determined changes both in the network of relations binding individuals to the people who are close in everyday life and in the availability of tangible and intangible resources they exchange. Results from surveys carried out in several countries during the hardest lockdown periods reported, in general, good and positive relationships inside the household environment when forced to stay at home, and a rise of “virtual” relations with non-cohabiting people. On the one hand, perceiving the household environment as very good has helped people live positively and constructively, highlighting the importance of family ties. On the other hand, the lack of cohabitants—together with containment measures—may have worsened situations of loneliness or raised the perception of need among people living alone. The lack of physical sociability (meeting and spending time together) can have compromised the instrumental support, meaning material resources, services, and tangible help. This loss may be serious for those who were used to receiving instrumental support through a physical presence, such as young parents who often count on grandparents for the care of children or elders who need for daily care and social companionship. For elders living alone, the lack of physical and immediate support can be hard to manage, and this condition can worsen the poor ability or habit to use smartphones, tablets, or personal computers for video calls and chatting.

The strong reduction of inter-generational physical contacts observed during the lockdown among younger adults—probably due to the wish to avoid contacts by grandchildren with older people—is a new behavior to consider and monitor. From one side, this attitude can be health-protective for the elderly, but from another side, it carries the risk of leaving the elderly even more alone. The containment measures that have reduced the possibility to travel, together with the suspension of health services, further undermines the condition of frail individuals.

From the short review on several surveys carried out in Italy to study relational aspects during the SARS-CoV2 pandemic, we recognize that these surveys provided several relevant and new insights into the study of the meso-level of the pandemic situation. Nevertheless, the characteristics of these surveys show at least two drawbacks: the sample size does not allow us to describe detailed behaviors of specific groups of people, and these surveys are not included in the National Statistical Programme (NSP), which establishes the statistical surveys of public interest. The first aspect could hinder an analytical procedure for identifying and then monitoring relationally vulnerable individuals. The exceptional character of these surveys and lack of acknowledgement of an official survey from the NSP does not ensure future editions. This would hinder the comparison of an emergency with a normal situation, limiting their use for comparative analyses by periods and territorial areas.

This paper aimed to contribute to the existing discussion on the consequences of the containment measures by focusing on two specific groups—young adults and the elderly—often recognized in the literature as more vulnerable than other age groups. Using the most recent available Italian data of the FSS 2016, we built the ego-centered networks of young adults and the elderly in light of COVID-19 containment measures. FSS data are the only data that mimic a credible relational context in the light of social distancing limitations. Despite the four-year lag with respect to the outbreak of the SARS-CoV-2 virus, from these data, we propose a frame to identify in advance—and eventually to protect by the adoption of effective strategies and interventions at the local level—groups of individuals vulnerable with respect to social relations and exchange of resources (relational vulnerability). To study the networks people would count on under social distancing restrictions, the “easy-to-reach” network has been defined considering only alters reachable inside the borders of the municipality of residence of the respondents, defining the physical proximity of alters. In the “accustomed-to-reach” network, a further constraint has been added with respect to the frequency of face-to-face contacts. The rationale behind the “easy-to-reach” and the “accustomed-to-reach” network construction is that it is more likely that the “easy-to-reach” network can be an ultimate resource in case of need, while the “accustomed-to-reach” network represents a primary source of support in situations of reduced mobility (e.g., a new lockdown).

The two most common “easy-to-reach” network types shared by young adults are either the *Non-kin*, driven by the presence of friends, or the *Mixed*, with friends and/or neighbors coupled with alters in the family. Focusing on the elderly population, most people are embedded in either a *Kin* or a *Mixed* types, denoting the constant presence of non-cohabiting family members in the set of alters. Some differences can be noted in young adults, both single and in couple, with singles on average having more friends living nearby than young adults in couple. Looking at the elderly, although the average number of friends living nearby drops consistently both for females and males, slight differences can be observed in *Non-kin* types for single males with respect to males in couple. The magnitude of isolated individuals (in the *No Alters* network type) is over 10% in both groups, with a minimum value of 7.5% for single young females and a maximum of 14.6% in the case of the single elderly males.

The “accustomed-to-reach” network highlights not only the physical presence of alters, but also the habit of meeting and spending time together. In these networks, the frequency of physical contacts with siblings drops in all considered groups. The major difference with respect to the “easy-to-reach” network definition is the increase in the percentage of people, of both age groups, with no alters. In particular, the percentage of elder single males that are not only physically, but also socially isolated has been rising from 14.6% (in case of “easy-to-reach” network) to 17.6% (in case of “accustomed-to-reach” network).

The approach used for constructing personal networks allows us to bring in the foreground people exposed to relational vulnerability. These are as the *No Alters* because they declared in the survey not having external people to share activities and resources. Considering living arrangement, stage of life course, and type of network, we then suggest a distinction of individuals into three level of relational vulnerability. The analysis of the most vulnerable *No Alters* individuals by age, gender, and context of residence revealed that to be single is often associate with a condition of relational vulnerability not only among elderly people—especially for females over 75—but also for young adults, especially if aged between 25 and 34.

The results highlight the role of context of residence, measured both by territorial area and place of residence. The presence of individuals at the three levels of relational vulnerability does not change between territorial areas, but if we focus on *No Alters* individuals living in metropolitan areas, we observed a lower percentage of elder females and single young adults males in conditions of relational vulnerability. This protective effect with respect to the isolation of metropolitan areas could be explained by the scarce statistical representativeness of the sample data, calling for more adequate information to evaluate the role played by context of residence in relational vulnerability. Nevertheless a metropolitan area can be seen as an interesting place of residence to consider for monitoring and facing a risk situation. From one side, the higher number of inhabitants can bring in the foreground a wider and more diverse set of case studies. From the other side, in these places, more public services are available for citizens, but at the same time individuals can feel more alone and have fewer ties in comparison with small municipalities, where habits, life, and distance can facilitate social interaction. With respect to education, the highest level of relational vulnerability (very critical) is associated with a low education level, especially for the elderly.

We are aware that our analysis on relational vulnerability focused only on individuals who did not have any alters in their personal networks, under the assumptions of the proposed network definitions. We made this choice based on the theoretical identification of the basic elements of relational vulnerability and the available data from the FSS 2016 survey, taking into account the most recent months of COVID-19 restrictions with respect to physical distancing. In a different situation, the assumption on residential proximity of the kin alters living in the same municipality could be relaxed, allowing for alters who reside in another municipality but, for instance, not further away than 16 km, to be reliable potential supporters. In this case, based on the network construction, the *No Alters* type would be less frequent in the “easy-to-reach” and the “accustomed-to reach” networks, while the other network types would be more frequent.^17^ Additionally, the numbers of alters in the other ego-network types would increase. This would mean an increase in potential supporters.

The focus on *No Alters* individuals has been proposed also with the aim of providing operative suggestions for adding information on relational context in which the individuals are embedded when local public administrations projects monitor and manage at-risk situations. This should bring a more widespread data collection awareness with regard to personal networks.

Furthermore, we are aware of the limitations in the network measurement adopted in FSS questionnaires. Measuring social relations and social support involves many dimensions, but unfortunately, available data do not provide information to deeply investigate issues related to the type of relations entertained with different alters and to the residential proximity of friends. In addition, other important aspects related to relational vulnerability (e.g., the degree of dissatisfaction with available support; the perception of loneliness or of a state of need) are not detected in the survey.

We are also aware that the size and composition of alters is not sufficient to capture all the facets related to relationships. Nevertheless, they provide useful suggestions on the availability of resources, especially material or instrumental resources (e.g., medical care, adult assistance, providing meals) that can satisfy primary needs during a pandemic.

## Data Availability

The datasets generated and/or analyzed during the current study are available in the Istat repository, https://www.istat.it/it/archivio/237448.

## Footnotes

1 Dpcm 23 febbraio: https://www.gazzettaufficiale.it/eli/id/2020/02/23/20A01228/; dpcm 25 febbraio: https://www.gazzettaufficiale.it/eli/id/2020/02/25/20A01278/; dpcm 1 marzo: http://www.governo.it/it/articolo/coronavirus-firmato-il-dpcm-1-marzo-2020/14210; dpcm 4 marzo: http://www.governo.it/it/articolo/coronavirus-firmato-il-dpcm-4-marzo-2020/14241; dpcm 8 marzo: http://www.governo.it/it/articolo/coronavirus-firmato-il-dpcm-8-marzo-2020/14266; dpcm 11 marzo: http://www.governo.it/it/articolo/coronavirus-conte-firma-il-dpcm-11-marzo-2020/14299.

2 In the last decade the percentage of people aged 65-74 using personal computers every day has been increasing up to 18.8%, and the percentage of people aged 65 or older using the internet every day also increased up to 17.4% in 2019. Similar patterns are observed among young people (18-34 ys.), except for the use of personal computers, which has slightly decreased since 2013 to an average value of 43.1% in 2019. The percentage of people aged 25-44 using the internet every day increased up to 89.1% in 2019 (our elaboration from data in I.Stat: http://dati.istat.it/).

3 The last detailed data on the frequency and type of digital contacts refer to four years ago (see "Ego-centered network approach to studying social relations").

4 Recent Italian data on behaviors and opinions during the second wave of the pandemic (December 2020–January 2021) confirms the positive familiar environment, and show a strong reduction in meetings with both non-cohabiting family members and friends. The contacts by phone helped keep family and friendship relationships alive. The friendship and neighbor networks have proved to be more solid and supportive for young people than for over-65s (Istat, 2021).

5 Usually multipurpose surveys on population, e.g. European Quality of Life Survey—https://www.eurofound.europa.eu/surveys/european-quality-of-life-surveys/european-quality-of-life-survey-2016—International Social Survey Programme—http://w.issp.org—Generations and Gender Surveys—https://www.ggp-i.org/data/—or surveys addressed to specific age groups such as the Survey of Health, Ageing and Retirement in Europe—http://www.share-project.org/—devoted to individuals aged 50 and over.

6 The answer categories are as follows: every day, a few times a week, once a week, a few times a month but fewer than 4, a few times a year, never.

7 The answer categories are as follows: in another apartment of the same building, in the same municipality, in another municipality of Italy—less than 16 km, from 16 to 50 km, more than 50 km, abroad.

8 In all, 10% of singles and 2% of partners in couples are students.

9 Since the available data did not collect information on the potential role of the partner in providing support, cohabiting partners were not considered in the network of the partners in a couple, albeit their important role.

10 Unfortunately, the FSS questionnaire does not provide sufficient information to make any assumption on the residential proximity of other relatives.

11 Individuals that declare the presence of at least one not-cohabitant alter could potentially be in a condition of relational vulnerability as well. However, this condition may not be due to the lack of external people, but rather to dissatisfaction with the available relations, with the support received, and with the perception of loneliness. This information is not available in the FSS survey.

12 The FSS question on neighbors asks for the presence of neighbors (at least one) without reporting their precise number. In the network size computation, we consider one alter if the respondent declared to have neighbors, thus reporting only the median in Table 3.

13 The hypotheses on friends and neighbors did not change with respect to the “easy-to-reach” network.

14 In the set of figures, the percentages refer to the subgroups of individuals divided by gender inside each category of “very critical” and “more critical” *No Alters* respondents.

15 The complementary percentages represent respondents who are living in municipalities up to 10,000 inhabitants or beyond 10,000.

16 From Table 1, we can only know that more than 75% of the two age groups are employed or pensioners.

17 More specifically, the percentages of the No Alters type would be reduced to the values of 5% and 8% for young adults and the elderly, respectively, with no differences by living arrangements.
